# Acute hot‐water immersion augments the diastolic blood pressure nocturnal dip in healthy adults

**DOI:** 10.1113/EP093583

**Published:** 2026-03-09

**Authors:** Samuel F. Leaney, Ferrida A. F. Ponce, Tomos F. Owen, Benjamin L. Harvey, Oliver N. Davies‐Wilson, Oliver M. A. Brand, Geoff B. Coombs, Jonathan P. Moore, Samuel J. Oliver

**Affiliations:** ^1^ Institute for Applied Human Physiology, School of Psychology and Sport Science, College of Medicine and Health Bangor University Bangor UK

**Keywords:** ambulatory blood pressure, heat stress, heat therapy

## Abstract

Hot‐water immersion (HWI) has been shown to reduce 24 h ambulatory systolic blood pressure in hypertensive adults and might represent a preventative strategy for maintaining cardiovascular health in normotensive adults. The purpose of this study was to determine the time course of post‐HWI hypotension and test the hypothesis that a single HWI reduces subsequent 24 h ambulatory blood pressure (ABP) in healthy adults. In a randomized, crossover design, 23 participants [7 female and 16 male; 26 (4) years of age] underwent blood pressure assessments before, during, immediately after and for 24 h following 60 min of immersion in 40.4°C hot water (HWI) and a thermoneutral (24.7°C) air control (CON). Thermal and cardiovascular variables were assessed during and for 60 min after the intervention, after which the participants were instrumented with an ABP monitor for 24 h. At min 60 of the interventions, body core temperature was higher [CON, 36.93 (0.28)°C; HWI, 38.83 (0.19)°C; *P* < 0.001] and diastolic (*P *< 0.001) and mean (*P *< 0.001) arterial blood pressure lower in HWI than CON. HWI increased the diastolic blood pressure nocturnal dip compared with CON [CON, 15.4 (7.2)%; HWI, 19.1 (7.4)%; *P* = 0.022]. No differences in ABP were observed for 24 h, daytime or nighttime systolic, diastolic or mean arterial blood pressures (all *P *> 0.05). In conclusion, HWI transiently decreased mean arterial blood pressure and diastolic blood pressure for ≤20 min post‐heating and increased the subsequent nocturnal diastolic blood pressure dip in healthy adults.

## Abstract (highlights)

- **What is the central question of this study?**
  Is arterial blood pressure reduced in the 24 h following a single morning session of hot‐water immersion, when assessed by ambulatory blood pressure monitoring in healthy adults?
- **What is the main finding and its importance?**
  Morning hot water‐immersion lowered mean arterial and diastolic blood pressure during and immediately after the hot‐water immersion and increased the nocturnal diastolic blood pressure dip in healthy adults. Hot‐water immersion causes a hypotensive effect that influences nighttime blood pressure.

## 1 INTRODUCTION

Hypertension is a major risk factor for morbidity and mortality (The Global Cardiovascular Risk Consortium, 2023). Importantly, hypertension is a modifiable risk factor that can be prevented through healthy lifestyle alterations, such as physical activity (Barone Gibbs et al., 2021; Jones et al., 2025). However, approximately one in three adults globally (31.3%; 1.8 billion) do not meet physical activity guidelines (Strain et al., 2024). Thus, efforts have been made to investigate the efficacy of physical activity alternatives for hypotensive effects, including passive heat therapy, such as hot‐water immersion (HWI). Although heat therapy might not mimic all the beneficial effects of exercise (Cullen et al., 2020), for those unable or unwilling to exercise, heat therapy might be an alternative or adjunct to exercise to help maintain normal blood pressure (Pizzey et al., 2021). In fact, research suggests the specific hypotensive effects of exercise in conjunction with heat therapy might be greater than the effects of regular exercise alone (Akerman et al., 2019; Roxburgh et al., 2023) or exercise with thermoneutral water immersion (Steward et al., 2025).

Similar to postexercise hypotension (Wegmann et al., 2018), recent evidence indicates that the magnitude of the blood pressure drop immediately after HWI and light exercise is associated with longer‐term reductions in resting blood pressure, with larger immediate post‐HWI reductions being associated with greater reductions in resting blood pressure (Roxburgh et al., 2023). Systolic blood pressure was also recently shown to be lower in a hypertensive population across 24 h following HWI (Roxburgh et al., 2025). This profound and persistent post‐HWI hypotension might offer a window of reduced haemodynamic load and, if performed regularly, could be an important stimulus and mechanism to explain the observed cardiovascular benefits of regular HWI (Brunt & Minon, 2021; Cheng & MacDonald, 2019). However, whether persistent reductions in blood pressure (i.e. over 24 h) occur in healthy adults is unknown, but might be expected because reductions in blood pressure are observed after regular HWI in healthy and hypertensive adults (Brunt et al., 2016; Pizzey et al., 2021).

Ambulatory blood pressure (ABP) provides a comprehensive assessment of blood pressure in free‐living individuals and is the most robust and clinically prognostic assessment of blood pressure (Pickering et al., 2006; Yang et al., 2019). Additionally, 24 h ABP provides new insight into the diurnal changes in blood pressure between day and nighttime (e.g. nocturnal dip). The prognostic value of nighttime blood pressure might exceed that of daytime values alone (Hansen et al., 2011), with the European Society of Hypertension recommending that nighttime and nocturnal dipping be assessed by 24 h ABP monitoring (Parati et al., 2025; Stergiou et al., 2021). Given the similar physiological effects of exercise and passive heating (Cullen et al., 2020) and given that exercise has been shown to enhance the nocturnal blood pressure dip (Ernst et al., 2023; Jones et al., 2009; Park et al., 2005; Wuerzner et al., 2013), HWI might also be expected to enhance nocturnal blood pressure in the 24 h window post‐HWI.

Determining the effect of HWI on 24 h, daytime and nighttime blood pressure in healthy adults is an essential first step towards elucidating the time course of blood pressure changes, establishing the efficacy of HWI to reduce blood pressure and providing further insight into the possible mechanisms to explain the long‐term cardiovascular health benefits of heat therapy. Therefore, the purpose of this study was to determine the time course of post‐HWI hypotension and test the hypothesis that a single HWI reduces subsequent 24 h ABP in healthy adults. Specifically, we hypothesized that 24 h systolic blood pressure and nighttime blood pressure would be lower and the nocturnal dip greater after HWI than a time‐of‐day matched control in ambient room air.

## 2 MATERIALS AND METHODS

### 2.1 Ethical approval and informed consent

This study was approved by the School of Psychology and Sport Science Academic Research Ethics committee, Bangor University (#2024‐0257‐1), and all procedures complied with the *Declaration of Helsinki*, except for registration in a database. Written informed consent was obtained from all participants before enrolment.

### 2.2 Participants

Twenty‐three participants were recruited for the study [7 females and 16 males; 26 (4) years of age, 175 (9) cm tall, with body mass 75 (16) kg, body fat 17.0 (6.9)% and physical activity 5111 (4844)  MET min week^−1^]. An a priori sample size estimation (α = 0.05 and power = 0.80) determined that a total of 22 participants would be adequate to detect a 3.9 mmHg reduction in mean 24 h SBP (effect size, *dz *= 0.63) using Student's two‐tailed, dependent *t*‐test (G*Power v.3.1.9.4: Heinrich Heine University Düsseldorf, Düsseldorf, Germany). The sample size calculation was based on 24 h ABP pilot data collected in our laboratory (*n* = 7), which indicated that the 24 h mean SBP in young healthy adults was 119 mmHg, with a pooled SD of 8 mmHg and a correlation of *r* = 0.7 for between‐day measurements. The 3.9 mmHg reduction in SBP was chosen because it has previously been observed in a meta‐analysis as the mean change in SBP following longer‐term heat therapy (Pizzey et al., 2021). Moreover, following a single hot‐water immersion, Roxburgh et al. (2025) demonstrated a 7 mmHg reduction in 24 h mean SBP (*dz* = 1.06) in a hypertensive population. We selected the smaller 3.9 mmHg reduction (*dz* = 0.63) in SBP because our population consisted of healthy adults, who might be expected to have more conservative reductions in ABP following heat stress (Pizzey et al., 2021).

Participants were healthy adults 21–34 years of age; they were non‐smokers and free from known cardiovascular, neurological, respiratory or metabolic disease. Participants also had no history of heat illness and were not engaged in any form of regular passive heat therapy, i.e., hot‐water immersion or sauna, more than once per week. To account for fluctuations in body temperature (Baker et al., 2020) and vascular function (Williams et al., 2020), female participants were studied in the first 7 days of their early follicular phase.

### 2.3 Study design

**FIGURE 1:**
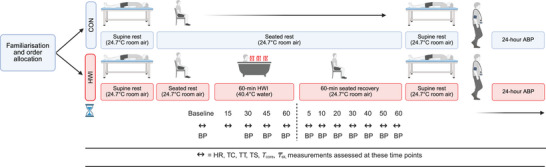
Experimental protocol. Abbreviations: ABP, ambulatory blood pressure; BP, blood pressure; CON, control trial; HR, heart rate; HWI, hot‐water immersion trial; TC, thermal comfort; *T*_core_, core body temperature; TS, thermal sensation; *₸*_sk_, mean skin temperature; TT, thermal tolerance. Created with BioRender.com.

Participants visited the laboratory on three occasions, once for familiarization and subsequently for two experimental trials. A counterbalanced crossover design was used, with each participant completing both experimental trials separated by ≥48 h. Experimental trials consisted of either 60 min of HWI or a time‐of‐day matched control in ambient room air (CON). Participants completed the two experimental trials in the morning at the same time of day (∼09.00 h) and followed the same experimental procedures (Figure 1).

### 2.4 Familiarization

Before the two experimental visits, a familiarization session was conducted to accustom participants to the laboratory environment and experimental trials. During this visit, the height of participants was determined by a wall‐mounted measuring tape, and body mass and composition by bio‐electrical impedance analysis weighing scales (BC‐418 MA, Tanita, Manchester, UK). Physical activity was assessed by the International Physical Activity Questionnaire long form (Craig et al., 2003), which provides a self‐reported assessment of physical activity across job, transport, domestic and leisure, including walking, moderate and vigorous activity.

### 2.5 Experimental procedures

For experimental trials, participants arrived having refrained from heat exposure for ≥36 h, strenuous exercise for ≥24 h, alcohol and caffeine for ≥12 h and food for ≥6 h. Before experimental trials began, participants provided a small urine sample (<25 mL) to confirm their hydration status with a urine osmolality reader (Pocket PAL‐OSMO, Vitech Scientific, Japan) and recorded nude body mass after voiding their bladder.

To begin, participants were instrumented for physiological monitoring. Heart rate was recorded with a wireless heart rate monitor (Polar H7 0B, Polar Electro Oy, Kempele, Finland) and brachial blood pressure by a validated brachial cuff‐based automated oscillometric blood pressure monitor (UM‐211, A & D Medical, Abingdon, UK) (Fania et al., 2017). In accordance with the European Society of Hypertension practice guidelines (Stergiou et al., 2021), blood pressure measurements were taken seated, with the participant's back supported by a chair, legs uncrossed, feet flat on the floor, with their arm resting at heart level, with the mean of the last two of three measurements used to calculate SBP and diastolic blood pressure (DBP). Mean arterial pressure (MAP) was calculated using the equation:

$$\mathrm{MAP}=\left(1/3\times \mathrm{SBP}\right)+\left(2/3\times \mathrm{DBP}\right)$$

Rate–pressure product (RPP) and pulse pressure (PP) were calculated as follows:

RPP=HR×SBP

PP=SBP−DBP

Body core temperature (*T*_core_) and skin temperature were measured by a rectal thermistor self‐inserted 15 cm beyond the anal sphincter (YSI 4000A, Dayton, OH, USA) and skin thermistors on the thigh, calf, chest and upper arm (2020 Series, Grant Instruments, Royston, UK). Mean skin temperature (*₸*_sk_) was calculated using the following equation (Ramanathan, 1964):

$${\bar{T}}_{\mathrm{sk}}=0.3\left(T_{\mathrm{chest}}+T_{\mathrm{arm}}\right)+0.2\left(T_{\mathrm{thigh}}+T_{\mathrm{calf}}\right)$$

Mean body temperature (*₸*_b_) was calculated using the following equation (Burton, 1935; Lenhardt & Sessler, 2006):

$${\bar{T}}_{b}=\left(0.64\times T_{\mathrm{core}}\right)+\left(0.36\times {\bar{T}}_{\mathrm{sk}}\right)$$

### 2.6 Experimental interventions (HWI and CON)

During the HWI trial, participants wearing swimsuits entered a prefilled hot bath (LUMI recovery PRO) that was set and maintained at 40.4 (0.4)°C. Participants positioned themselves in a semi‐recumbent position to ensure the water level reached their neck with arms under the water for 30 min; thereafter, participants were seated upright in waist‐deep water, with arms resting at heart level out of the water, for a further 30 min. Participants were immersed for a total of 60 min. During the CON trial, dressed in shorts and a t‐shirt, participants rested in a seated position in a temperate laboratory [temperature 24.7 (1.8)°C]. Participants were allowed to drink water ad libitum and were provided with water to consume after HWI to replace fluid losses. The *T*_core_, *₸*_sk_, heart rate, and thermal perception by standardized scales (International Organization for Standardization, 2019) were quantified at baseline (pre‐immersion), at 15, 30, 45 and 60 min during the HWI or CON interventions (time points: 15, 30, 45 and 60 min), whilst blood pressures were quantified at baseline (pre‐immersion) and at 30, 45 and 60 min during the HWI or CON interventions (time points: 30, 45 and 60 min). Immediately afterwards, *T*_core_, *₸*_sk_, heart rate, blood pressure and thermal perception were quantified at 5, 10, 20, 30, 40, 50 and 60 min after CON and HWI. Post‐trial nude body mass was measured before voiding the bladder.

At the end of the experimental trials, participants were instrumented with a validated brachial cuff‐based oscillometric, ABP monitor (ABPM, Mobil‐O‐Graph, IEM, Aachen, Germany). In line with the European Society of Hypertension practice guidelines for ABP (Parati et al., 2014), care was taken to fit participants with an appropriately sized cuff on the non‐dominant arm, and 24 h ABP was recorded once every 20 min during daytime hours (∼08.00–22.00 h) and once every 30 min during nighttime hours (∼22.00– 08.00 h). Participants were provided with printed instructions for ABP measurement standardization, which included the avoidance of exercise, caffeine and alcohol consumption. Participants were asked to log their food intake during the 24 h recording period and replicate their food intake in their second trial as closely as possible using the food log from their first trial. Participants recorded the time of sleep and waking with a self‐reported sleep diary and were instrumented with a pedometer (SW200 Digi‐Walker, Yamax, Tokyo, Japan) to calculate step count.

Hourly averaged blood pressure (SBP, DBP and MAP) was used to calculate mean overall, daytime and nighttime blood pressures. In accordance with the European Society for Hypertension practice guidelines (Parati et al., 2014; Stergiou et al., 2021), daytime and nighttime periods were defined by participants’ self‐reported sleep times, or when absent (*n* = 1) were defined as fixed time periods (daytime 08.00–20.00 h and nighttime 01.00– 06.00h). Nocturnal blood pressure dip percentage was calculated using the following equation:

$$\mathrm{Nocturnal}\;\mathrm{dip}\;\left(\%\right)=\frac{\left(\mathrm{Daytime}\;-\;\mathrm{nightime}\right)}{\mathrm{Daytime}}\times 100$$

### 2.7 Statistical analysis

Statistical analysis was conducted using IBM SPSS Statistics v.29 (IBM Corp., Armonk, NY, USA), and figures were created with GraphPad Prism v.10 (Prism, San Diego, CA, USA). Local average of the nearest neighbour mean imputation was used to replace missing time‐series values during the interventions and 60 min recovery (Flores et al., 2019). Values are the mean (SD) unless otherwise stated, and statistical significance was set at *P* < 0.05. To determine differences in thermal, cardiovascular and perceptual data during HWI and CON interventions, a two‐way ANOVA was used with trials (HWI and CON) and time points (pre‐immersion baseline, 15, 30, 45 and 60 min). To determine differences in responses post‐HWI, a two‐way ANOVA was used with trials (HWI and CON) and time points (pre‐immersion baseline and 5, 10, 20, 30, 40, 50 and 60 min recovery). Where an interaction effect was observed (trial × time), *post hoc* Bonferroni correction for multiple comparisons was performed. Two‐way ANOVA effect sizes, partial eta squared ($\eta_{P}^{2}$) were used and interpreted as small if <0.06, medium if 0.06–0.14 and large if >0.14. Student's paired samples, two‐tailed *t*‐tests were conducted to compare CON and HWI trials on pre‐intervention baseline thermal, cardiovascular, urine osmolality, fluid losses and changes in nude body mass, in addition to all ABP outcomes. For Student's paired samples *t*‐test effect sizes, Cohen's *d* was used, where *d* values of 0.2, 0.5, and 0.8 were interpreted as small, medium and large, respectively.

## 3 RESULTS

All 23 participants completed both experimental trials. Although there were some reports of light‐headedness during HWI, no adverse events were reported, and all participants completed the full immersion protocol. One female participant was excluded from ABP analysis because they were unable to follow experimental instructions during one of the trials during the 24 h ABP measurement. Another female participant was excluded from skin and mean body temperature analysis owing to equipment malfunction.

### 3.1 Thermal, cardiovascular and perceptual responses to CON and HWI

There was no difference in baseline urine osmolality at the start of experimental trials [CON, 629 (246) mO smol kg^−1^; HWI, 603 (265) mOsmol kg^−1^; *P* = 0.568], and pre‐intervention (baseline) thermal, cardiovascular and perceptual measures were not different between CON and HWI (all *P *> 0.05; Table 1). During HWI, *T*_core_, *₸*_sk_, *₸*_b_, heart rate, PP, RPP and thermal sensation increased, whereas thermal comfort and thermal tolerance decreased (interaction effect, all *P *< 0.001; Table 1). DBP and MAP were lower during HWI in comparison to CON (interaction effect *P *< 0.001; Table 1). SBP decreased with time irrespective of experimental trial (time effect *P* = 0.022; Table 1), but there was no interaction (*P* = 0.440).

**TABLE 1:** Thermal, cardiovascular and perceptual responses to 60 min in 24.7°C air (CON) and 40.4°C hot‐water immersion (HWI).

			Intervention						
Variable	Trial	Baseline	15 min	30 min	45 min	60 min	*P* ($\eta_{P}^{2}$)		
							Trial	Time	Trial × time
*T*_core_, °C	CON	37.04 (0.24)	37.00 (0.30)	36.99 (0.26)	36.95 (0.29)	36.93 (0.28)*	<0.001 (0.966)	<0.001 (0.938)	<0.001 (0.939)
	HWI	37.13 (0.26)	37.56 (0.31)*^#^	38.45 (0.31)*^#^	38.74 (0.22)*^#^	38.83 (0.19)*^#^			
*₸*_sk_, °C	CON	31.98 (1.06)	31.90 (0.95)	31.98 (0.92)	31.89 (0.92)	31.91 (0.91)	<0.001 (0.975)	<0.001 (0.931)	<0.001 (0.943)
	HWI	31.71 (0.97)	39.23 (0.66)*^#^	38.14 (1.06)*^#^	36.93 (0.36)*^#^	36.92 (0.49)*^#^			
*₸*_b_, °C	CON	35.22 (0.37)	35.16 (0.35)	35.19 (0.35)	35.13 (0.38)	35.12 (0.37)	<0.001 (0.989)	<0.001 (0.957)	<0.001 (0.964)
	HWI	35.18 (0.38)	38.13 (0.42)*^#^	38.34 (0.43)*^#^	38.09 (0.22)*^#^	38.14 (0.26)*^#^			
Heart rate, beats min^−1^	CON	69 (11)	67 (9)	64 (8)*	65 (8)*	65 (8)	<0.001 (0.932)	<0.001 (0.787)	<0.001 (0.873)
	HWI	70 (9)	99 (13)*^#^	113 (10)*^#^	104 (11)*^#^	103 (11)*^#^			
SBP, mmHg	CON	108 (8)	–	107 (9)	107 (7)	108 (9)	0.728 (0.006)	0.022 (0.134)	0.440 (0.040)
	HWI	109 (6)	–	105 (10)	106 (8)	108 (8)			
DBP, mmHg	CON	70 (7)	–	69 (7)	70 (6)	71 (6)	<0.001 (0.787)	<0.001 (0.739)	<0.001 (0.654)
	HWI	69 (7)	–	53 (5)*^#^	53 (5)*^#^	53 (6)*^#^			
MAP, mmHg	CON	82 (6)	–	81 (6)	82 (6)	83 (6)	<0.001 (0.724)	<0.001 (0.671)	<0.001 (0.588)
	HWI	83 (6)	–	70 (5)*^#^	71 (5)*^#^	71 (6)*^#^			
PP, mmHg	CON	38 (9)	–	38 (8)	37 (7)	37 (8)	<0.001 (0.691)	<0.001 (0.563)	<0.001 (0.496)
	HWI	39 (6)	–	52 (10)*^#^	53 (8)*^#^	55 (8)*^#^			
RPP, mmHg	CON	7401 (1326)	–	6869 (988)*	6903 (785)	7027 (923)	<0.001 (0.877)	<0.001 (0.717)	<0.001 (0.833)
	HWI	7590 (1000)	–	11 921 (1548)*^#^	11 088 (1633)*^#^	11 097 (1696)*^#^			
Thermal comfort	CON	0.0 (0.1)	0.0 (0.2)	0.0 (0.2)	0.0 (0.0)	0.0 (0.0)	<0.001 (0.818)	<0.001 (0.550)	<0.001 (0.551)
	HWI	0.1 (0.5)	1.4 (0.7)*^#^	2.2 (0.9)*^#^	1.6 (1.0)*^#^	1.2 (1.0)*^#^			
Thermal sensation	CON	0.0 (0.8)	0.0 (0.5)	0.1 (0.4)	0.0 (0.5)	0.0 (0.5)	<0.001 (0.884)	<0.001 (0.653)	<0.001 (0.633)
	HWI	0.3 (0.7)	2.3 (0.8)*^#^	2.9 (1.1)*^#^	2.3 (1.0)*^#^	2.2 (0.9)*^#^			
Thermal tolerance	CON	0.0 (0.0)	0.0 (0.0)	0.0 (0.0)	0.0 (0.0)	0.0 (0.0)	<0.001 (0.749)	<0.001 (0.476)	<0.001 (0.479)
	HWI	0.0 (0.0)	1.1 (0.8)*^#^	1.9 (1.1)*^#^	1.1 (1.0)*^#^	1.0 (1.0)*^#^			

*Note*: Data are presented as the mean (SD). *n *= 23 for all data except *₸*_sk_ and *₸*_b_ (*n *= 22). Data were collected before and during 60 min in 24.7°C air (CON) and 40.4°C hot‐water immersion (HWI). Thermal comfort ranges from 0, comfortable to 4, extremely uncomfortable; thermal sensation ranges from −5, extremely cold to 5, extremely hot; and thermal tolerance ranges from 0, tolerable to 4, intolerable. Results were analysed by two‐way ANOVA to assess the effect of trial (CON and HWI), time (baseline, 15, 30, 45 and 60 min) and interaction (trial × time). *Post hoc* tests were completed by Bonferroni‐corrected pairwise comparisons, where *P < 0.05 versus baseline within trial, ^#^ *P *< 0.05 versus respective CON time point. Abbreviations: DBP, diastolic blood pressure; MAP, mean arterial blood pressure; PP, pulse pressure; RPP, rate–pressure product; SBP, systolic blood pressure; *₸*_b_, mean body temperature; *T*_core_, core body temperature; *₸*_sk_, mean skin temperature.

Fluid losses across the interventions were higher in HWI than CON [CON, 0.15 (0.10) L; HWI, 1.01 (0.51) L; *P *< 0.001], but, owing to higher water intake in HWI compared with CON [CON, 0.13 (0.16) L; HWI, 0.99 (0.47) L; *P* < 0.001], there was no difference in nude body mass change across the trials [CON, −0.02 (0.17) kg; HWI, −0.03 (0.44) kg; *P* = 0.907].

After HWI, *T*_core_, *₸*_sk_, *₸*_b_, HR and RPP were higher than CON throughout the 60 min recovery (interaction effect *P *< 0.001; Table 2). MAP and DBP were lower than CON after HWI for 10 and 20 min, respectively, into seated recovery (Table 2). SBP was significantly higher at 10 min post‐HWI than CON (*P *= 0.019; Table 2). Thermal sensation was higher and thermal tolerance lower in HWI at 5 and 10 min into seated recovery compared with CON (Table 2). Thermal comfort was lower in HWI at 5 and 20 min into seated recovery compared with CON (Table 2).

**TABLE 2:** Thermal, cardiovascular and perceptual responses in seated recovery following 60 min in 24.7°C air (CON) and 40.4°C hot‐water immersion (HWI).

			Recovery									
Variable	Trial	Baseline	5 min	10 min	20 min	30 min	40 min	50 min	60 min	*P* ($\eta_{P}^{2}$)		
										Trial	Time	Trial × time
*T*_core_, °C	CON	37.04 (0.24)	36.91 (0.29)*	36.91 (0.30)*	36.90 (0.30)*	36.90 (0.30)	36.91 (0.28)	36.90 (0.29)	36.90 (0.30)	<0.001 (0.961)	<0.001 (0.911)	<0.001 (0.921)
	HWI	37.13 (0.26)	38.83 (0.19)*^#^	38.68 (0.21)*^#^	38.31 (0.26)*^#^	38.03 (0.28)*^#^	37.85 (0.28)*^#^	37.71 (0.33)*^#^	37.68 (0.32)*^#^			
*₸*_sk_, °C	CON	31.98 (1.06)	31.90 (0.87)	31.92 (0.86)	31.92 (0.88)	32.02 (0.89)	31.96 (0.92)	32.01 (0.93)	31.96 (0.96)	<0.001 (0.746)	<0.001 (0.719)	<0.001 (0.813)
	HWI	31.71 (0.97)	35.35 (0.86)*^#^	34.90 (0.80)*^#^	34.24 (0.83)*^#^	33.64 (0.92)*^#^	33.18 (0.86)*^#^	33.00 (0.88)*^#^	32.90 (0.90)*^#^			
*₸*_b_, °C	CON	35.22 (0.37)	35.11 (0.36)	35.11 (0.38)	35.11 (0.40)	35.15 (0.39)	35.13 (0.41)	35.14 (0.43)	35.13 (0.44)	<0.001 (0.921)	<0.001 (0.874)	<0.001 (0.929)
	HWI	35.18 (0.38)	37.58 (0.37)*^#^	37.32 (0.34)*^#^	36.85 (0.34)*^#^	36.46 (0.38)*^#^	36.18 (0.36)*^#^	36.02 (0.39)*^#^	35.97 (0.41)*^#^			
Heart rate, beats min^−1^	CON	69 (11)	64 (9)	64 (9)*	66 (8)	65 (8)	65 (8)	65 (10)	65 (8)	<0.001 (0.829)	<0.001 (0.572)	<0.001 (0.724)
	HWI	70 (9)	101 (15)*^#^	89 (13)*^#^	82 (10)*^#^	80 (9)*^#^	79 (10)*^#^	79 (8)*^#^	78 (9)*^#^			
SBP, mmHg	CON	108 (8)	109 (9)	108 (8)	107 (7)	108 (9)	107 (9)	109 (9)	107 (9)	0.792 (0.003)	<0.001 (0.151)	0.007 (0.118)
	HWI	109 (6)	109 (7)	112 (9)^#^	108 (8)	107 (8)	108 (9)	106 (8)	105 (9)			
DBP, mmHg	CON	70 (7)	70 (9)	71 (7)	71 (8)	72 (8)	72 (8)	72 (7)	72 (8)	0.003 (0.342)	<0.001 (0.442)	<0.001 (0.362)
	HWI	69 (7)	57 (8)*^#^	62 (8)* ^#^	67 (7) ^#^	67 (9)	69 (7)	69 (6)	68 (7)			
MAP, mmHg	CON	82 (6)	83 (8)	83 (7)	83 (7)	84 (7)	84 (7)	84 (7)	83 (7)	0.019 (0.225)	<0.001 (0.245)	<0.001 (0.223)
	HWI	83 (6)	74 (7)*^#^	79 (7)^#^	81 (6)	81 (8)	82 (7)	82 (6)	81 (7)			
PP, mmHg	CON	38 (9)	38 (7)	37 (7)	36 (8)	36 (9)	35 (8)	37 (8)	35 (9)	<0.001 (0.441)	<0.001 (0.617)	<0.001 (0.399)
	HWI	39 (6)	52 (8)*^#^	50 (9)*^#^	42 (7)^#^	39 (7)	39 (9)	37 (8)	37 (8)			
RPP, mmHg	CON	7401 (1326)	6976 (969)	6831 (874)	7007 (837)	6975 (867)	7002 (873)	6995 (938)	6902 (923)	<0.001 (0.802)	<0.001 (0.555)	<0.001 (0.675)
	HWI	7590 (1000)	10984 (1747)*^#^	10007 (1660)*^#^	8843 (1254)*^#^	8549 (1151)*^#^	8506 (1018)*^#^	8401 (870)*^#^	8166 (915)^#^			
Thermal Comfort	CON	0.0 (0.1)	0.0 (0.0)	0.0 (0.0)	0.0 (0.0)	0.0 (0.0)	0.0 (0.0)	0.0 (0.0)	0.0 (0.0)	0.004 (0.317)	<0.001 (0.158)	<0.001 (0.159)
	HWI	0.1 (0.5)	0.5 (0.6)^#^	0.2 (0.5)	0.2 (0.4)^#^	0.1 (0.3)	0.0 (0.1)	0.0 (0.1)	0.0 (0.1)			
Thermal Sensation	CON	0.0 (0.8)	0.0 (0.5)	0.0 (0.5)	0.0 (0.5)	0.1 (0.4)	0.1 (0.4)	0.1 (0.4)	0.1 (0.4)	0.013 (0.247)	<0.001 (0.166)	<0.001 (0.202)
	HWI	0.3 (0.7)	0.9 (0.8)^#^	0.5 (0.8)^#^	0.2 (0.6)	0.2 (0.6)	0.1 (0.4)	0.2 (0.4)	0.2 (0.4)			
Thermal Tolerance	CON	0.0 (0.0)	0.0 (0.0)	0.0 (0.0)	0.0 (0.0)	0.0 (0.0)	0.0 (0.0)	0.0 (0.0)	0.0 (0.0)	0.045 (0.171)	<0.001 (0.150)	<0.001 (0.153)
	HWI	0.0 (0.0)	0.3 (0.6)^#^	0.2 (0.4)^#^	0.1 (0.3)	0.0 (0.2)	0.0 (0.0)	0.0 (0.0)	0.0 (0.0)			

*Note*: Data are presented as the mean (SD). *n *= 23 for all data except *₸*_sk_ and *₸*_b_ (*n *= 22). Data were collected before (baseline pre‐intervention) and during 60 min recovery from 24.7°C air (CON) and 40.4°C hot‐water immersion (HWI). Thermal comfort ranges from 0, comfortable to 4, extremely uncomfortable; thermal sensation ranges from −5, extremely cold to 5, extremely hot; and thermal tolerance ranges from 0, tolerable to 4, intolerable. Analysis was by two‐way ANOVA to assess the effect of trial (CON and HWI), time (baseline, 5, 10, 20, 30, 40, 50 and 60 min) and interaction (trial × time). *Post hoc* tests were completed by Bonferroni‐corrected pairwise comparisons, where *P < 0.05 versus baseline within trial and ^#^ *P *< 0.05 versus respective CON time point. Abbreviations: DBP, diastolic blood pressure; MAP, mean arterial blood pressure; PP, pulse pressure; RPP, rate–pressure product; SBP, systolic blood pressure; *₸*_b_, mean body temperature; *T*_core_, body core temperature; *₸*_sk_, mean skin temperature.

### 3.2 Twenty‐four‐hour ABP after 60 min in CON and HWI

During the ABP monitoring, 99.1% of all data were collected, and all participants exceeded the minimum 70.0% data collection threshold for ABP (Parati et al., 2014). During the 24 h ABP monitoring, there were no differences between CON and HWI in physical activity [CON, 5611 (2880) steps; HWI, 6192 (2897) steps; *P* = 0.346] or self‐reported sleep duration [CON, 486 (82) min; HWI, 498 (81) min; *P* = 0.389].

**FIGURE 2:**
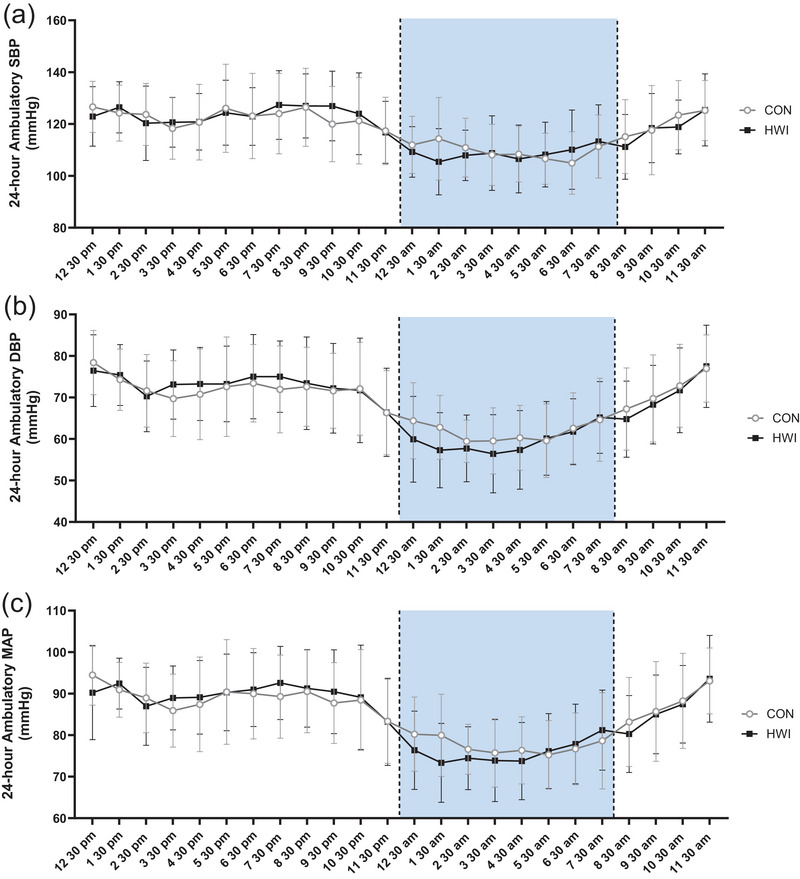
Twenty‐four‐four ambulatory systolic blood pressure (SBP; a), diastolic blood pressure (DBP; b) and mean arterial blood pressure (MAP; c) after 60 min in 24.7°C air (CON) and 40.4°C hot‐water immersion (HWI). Data are presented as the mean (CON, white circles; HWI, black squares) and SD (bars). The blue section denotes the mean nighttime period for participants across both trials. Data are *n* = 22 and presented to visualize the data set.

**FIGURE 3:**
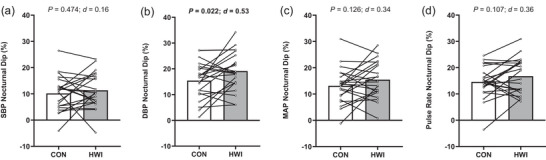
Systolic blood pressure (SBP) nocturnal dip percentage (a), diastolic blood pressure (DBP) nocturnal dip percentage (b), mean arterial blood pressure (MAP) nocturnal dip percentage (c) and pulse rate nocturnal dip percentage (d) measured during 24 h ambulatory blood pressure assessment after 60 min in 24.7°C air (CON) and 40.4°C hot‐water immersion (HWI). Mean values are indicated by white (CON) and grey (HWI) bars, respectively. White circles connected with black lines represent individual data. Analysed by Student's two‐tailed paired *t*‐tests. All data are *n* = 22.

The 24 h ABP data for SBP, DBP and MAP for CON and HWI are presented in Figure 2. No differences were observed between HWI and CON for 24 h, daytime or nighttime SBP, DBP, MAP or pulse rate (Table 3). The change in DBP pressure from daytime to nighttime, expressed as the nocturnal blood pressure dip, was greater following HWI than CON [CON, 15.4 (7.2)%; HWI, 19.1 (7.4)%, mean difference = 3.70, (confidence interval, CI = 0.59, 6.81); *P* = 0.022, *d* = 0.53; Figure 3b]. The nocturnal blood pressure dip was not different between CON and HWI for SBP [CON, 10.2 (6.7)%; HWI, 11.4 (6.7)%, mean difference = 1.16, (CI = − 2.15, 4.47); *P* = 0.474, *d* = 0.16; Figure 3a] or MAP [CON, 13.1 (6.8)%; HWI, 15.5 (6.9)%, mean difference = 2.34, (CI = −0.71, 5.38); *P* = 0.126, *d* = 0.34; Figure 3c]. The nocturnal pulse rate dip was not different between CON and HWI [CON, 14.6 (6.2)%; HWI, 16.8 (6.7)%, mean difference = 2.16, (CI = −0.51, 4.83); *P* = 0.107, *d* = 0.36; Figure 3d].

**TABLE 3:** Twenty‐four‐hour ABP after 60 min in 24.7°C air (CON) and 40.4°C hot‐water immersion (HWI).

Parameter	CON	HWI	Mean difference (95% CI)	*P*‐value	Cohen's *d*
**24 h ABP**					
**SBP, mmHg**	117.9 (8.3)	117.8 (8.0)	−0.11 (−1.56, 1.33)	0.873	0.04
**DBP, mmHg**	68.5 (4.9)	68.0 (6.0)	−0.53 (−2.04, 0.97)	0.469	0.16
**MAP, mmHg**	84.9 (5.2)	84.5 (5.7)	−0.33 (−1.72, 1.06)	0.624	0.11
**Pulse rate, beats min^−1^**	65 (7)	67 (7)	2.00 (−0.32, 4.32)	0.088	0.38
**Daytime ABP**					
**SBP, mmHg**	122.6 (10.3)	122.8 (8.9)	0.22 (−1.50, 1.93)	0.797	0.06
**DBP, mmHg**	72.7 (6.0)	73.1 (6.8)	0.43 (−0.95, 1.81)	0.526	0.14
**MAP, mmHg**	89.2 (6.6)	89.5 (6.6)	0.35 (−0.96, 1.66)	0.584	0.12
**Pulse rate, beats min^−1^**	69 (7)	71 (7)	2.59 (−0.28, 5.47)	0.075	0.40
**Nighttime ABP**					
**SBP, mmHg**	109.7 (7.7)	108.7 (9.7)	−0.97 (−4.43, 2.48)	0.565	0.13
**DBP, mmHg**	61.3 (4.4)	58.9 (5.9)	−2.33 (−4.71, 0.05)	0.055	0.43
**MAP, mmHg**	77.2 (4.9)	75.5 (6.3)	−1.68 (−4.29, 0.93)	0.194	0.29
**Pulse rate, beats min^−1^**	59 (7)	60 (8)	0.86 (−1.45, 3.18)	0.447	0.17

*Note*: Blood pressure data are presented as the mean (SD), with mean difference and 95% confidence intervals also presented. *n *= 22 for all data. Analysis was by Student's two‐tailed paired *t*‐tests. Abbreviations: ABP, ambulatory blood pressure; CI, confidence interval; DBP, diastolic blood pressure; MAP, mean arterial blood pressure; SBP, systolic blood pressure.

## 4 DISCUSSION

To our knowledge, this is the first study to examine the acute effects of HWI on 24 h ambulatory blood pressure in healthy adults. We observed that a single morning HWI significantly reduced MAP and DBP during and immediately after the immersion, indicating a clear transient hypotensive response. Although 24 h, daytime and nighttime ABP remained unchanged, the DBP nocturnal dip was greater during the night following HWI compared with the non‐immersion control. These new findings suggest that morning HWI might influence nocturnal blood pressure regulation, a factor linked to cardiovascular disease risk.

In the present study, SBP was well maintained during the HWI, whereas MAP was lower owing to a reduction in DBP, which is similar to previous observations (Gibbons et al., 2021; Leaney et al., 2025; Menzies et al., 2025; Steward et al., 2024). Reductions in arterial blood pressure are widely reported with elevations in body temperature and reflect reductions in systemic vascular resistance owing to cutaneous vasodilatation during heat stress (Crandall & González‐Alonso, 2010), despite increased sympathetic nerve activity (Low et al., 2011). The increase in cardiac output during dry heat stress is mediated through increases in heart rate, because stroke volume does not change appreciably (Minon et al., 1998; Wilson et al., 2009) or may even decrease (Foster et al., 2025) in healthy heat‐stressed participants. However, heat stress induced via HWI can increase cardiac output via elevated heart rate and stroke volume (Gibbons et al., 2021; Weston et al., 1987), presumably owing to the hydrostatic effects facilitating venous return and cardiac preload (Christie et al., 1990). Such an elevation in cardiac pressures might explain the maintenance of SBP during the immersion period. Consistent with this premise, RPP was increased during HWI compared with CON in the present study, which provides an indirect estimate of increased cardiac workload (Gobel et al., 1978; White, 1999).

During the post‐HWI recovery period, SBP, DBP and MAP were differentially affected (Table 2) such that SBP briefly increased, whereas DBP and MAP were initially decreased before returning to pre‐immersion baseline within 20 min (MAP) and 30 min (DBP). The DBP had the largest and most persistent post‐heating hypotension, with reductions of −12, −7 and −2 mmHg at 5, 10 and 20 min post‐HWI in comparison to the pre‐immersion baseline. These data are aligned with the recent meta‐analysis by Price et al. (2025), which indicated that a single heat therapy session reduces MAP and DBP, but not SBP. Price et al. (2025) reported that the mean DBP reduction immediately after HWI was 2 mmHg, which was also demonstrated for 20 min following HWI in our study. Importantly, in adults <50 years old, DBP provides additional prognostic value to SBP in predicting composite cardiovascular events and all‐cause mortality (Vishram‐Nielsen et al., 2021).

The 24 h rhythms in arterial blood pressure are influenced by endogenous circadian mechanisms and by 24 h behavioural and environmental factors, including sleep (Chellappa et al., 2019). ABP has improved reproducibility of mean blood pressure in comparison to in‐clinic blood pressure, which translates to better prediction of clinical outcomes and mortality and is the preferred method of non‐invasive 24 h blood pressure assessment (Mancia et al., 2023; Parati et al., 2025). To our knowledge, this is the first study to assess 24 h ABP responses following a single heat therapy session in healthy adults. In contrast to our hypothesis, HWI did not reduce 24 h mean, daytime mean or nighttime mean SBP, DBP or MAP compared with the non‐immersion control. This is probably attributable to the normotensive phenotype of our healthy participants, because the magnitude of hypotension after exercise and HWI is larger in people with higher pre‐immersion resting blood pressure. Indeed, reductions in 24 h ambulatory day and nighttime SBP have been observed in hypertensive adults across 24 h following a single HWI (Roxburgh et al., 2025).

A healthy reduction in blood pressure at night is between 10% and 20% of mean daytime values (Parati, 2000; Parati et al., 2025). Conversely, elevated nocturnal blood pressure and a lack of nocturnal dipping (<10%) are associated with an increased risk of end‐organ damage in hypertensive patients (Faraci & Scheer, 2024) and can contribute to atherosclerosis in both normotensive and hypertensive populations (Cuspidi et al., 2016). Our data demonstrate that a single HWI led to a greater DBP nocturnal dip, with a difference of 3.7%, between 15.4% in CON and 19.1% in HWI. In our study, although nighttime DBP was not statistically lower following HWI compared with CON, the mean difference in nighttime DBP was −2.33 mmHg (95% CI −4.71, 0.05), and the effect size was small to medium (*d* = 0.43 and *P *= 0.055), which might contribute to the DBP nocturnal dip being at the upper end of the healthy range (i.e. between 10% and 20%) (Parati, 2000; Parati et al., 2025). Although extreme dipping (>20%) might be deleterious only in older adults (>69 years old) (Palatini et al., 2020), the increase in nocturnal dip did not exceed this extreme dipping threshold (>20%).

The physiological mechanisms of nocturnal blood pressure regulation are multifactorial, and several regulatory systems have been proposed, including: (1) sodium and water handling; (2) the renin–angiotensin system; and (3) the autonomic nervous system (Parati et al., 2025). The mechanisms underlying the increased DBP nocturnal dip observed in the present study remain uncertain. Although most plasma volume expansion occurs within 24 h after heat exposure (Kissling et al., 2022), such early expansion would typically reduce supine blood volume redistribution and blunt baroreflex engagement, leading to a smaller nocturnal dip, yet we observed the opposite. In contrast, increased central blood volume at night might augment atrial stretch, stimulating atrial volume receptors and atrial natriuretic peptide secretion, promoting natriuresis /diuresis and suppression of vasopressin and renin–angiotensin–aldosterone activity, processes known to reduce circulating volume overnight (Dietz, 2005; Hoshide et al., 2021). The nocturnal nadir in cortisol might also contribute (Parati et al., 2025). However, because plasma volume, urine output and endocrine responses were not measured, this remains speculative. Future studies should quantify these variables to clarify mechanisms.

### 4.1 Methodological considerations

In addition to physiological variation, blood pressure responses are affected by postural factors. During HWI and 60 min recovery following HWI, blood pressure measurements were taken seated, with the participant's back supported by a chair, legs uncrossed, feet flat on the floor, with their arm resting at heart level. Although this standardization is in line with European Society of Hypertension practice guidelines (Stergiou et al., 2021), much of the physiological research assessing blood pressure in the recovery following HWI does so in the supine position (Amin et al., 2022; Francisco et al., 2021; Menzies et al., 2025). Differences in posture might affect the interpretation of our data, compared with that of previous work, but the implementation of standardized guidelines provides a stronger prognostic comparison.

Our control trial required participants to rest in ambient air rather than thermoneutral water because time was the main variable of interest to control. We also considered that an ambient control in dry air was a more suitable control, because thermoneutral water immersion is not a widely performed activity, and ambient air rest is representative of sedentary behaviour and provides a comparison to help answer the practical question of whether to adopt HWI or not. The addition of a thermoneutral water control would have enabled further insight into whether the observed change to nighttime blood pressure was related to heating or heating and hydrostatic effects, and might be an avenue for future research. Although HWI and thermoneutral water immersion have been shown to influence 24 h ABP (Roxburgh et al., 2025), 8 weeks of regular thermoneutral water immersion, in contrast to HWI, did not confer adaptations in cardiovascular function, including blood pressure (Brunt et al., 2016).

The use of ABP for the assessment of 24 h blood pressure will naturally incorporate variations in participants’ posture, mood, body temperature and activities of normal daily living across the 24 h measurement periods. In the present study, we attempted to limit these variations between trials by instructing volunteers to avoid alcohol and caffeine, replicate food intake and complete similar activities and sleep during each trial.

### 4.2 Applied implications and perspectives

Implementing regular exercise into daily routine is a primary strategy for the prevention of hypertension (Barone Gibbs et al., 2021; Jones et al., 2025), yet barriers such as low self‐esteem, low motivation or limited time hinder participation in regular exercise (Stutts, 2002). Our data demonstrate that a single session of morning HWI can elicit 24 h blood pressure modulation in normotensive individuals, particularly affecting nocturnal DBP, which is a phenomenon also seen following exercise (Ernst et al., 2023; Jones et al., 2009; Park et al., 2005; Wuerzner et al., 2013). Of note, DBP was affected to a larger magnitude than SBP during, immediately after, and across 24 h ABP in our study. Importantly, in young adults DBP provides additional prognostic value beyond SBP in predicting composite cardiovascular events and all‐cause mortality (Vishram‐Nielsen et al., 2021). This supports the concept that thermal therapy might have broader preventative applications beyond clinical treatment (Laukkanen et al., 2015; Ukai et al., 2020) and, therefore, might be an accessible alternative or an adjunct to exercise training (Cullen et al., 2020). Therefore, future research is warranted to explore whether regular HWI alone, or in combination with exercise, can produce cumulative or sustained benefits in 24 h ABP in healthy adults and those with disease. This future research will need to consider carefully the available literature when designing the HWI intervention, because 30 sessions over 8–10 weeks of HWI (at 40°C) for 45 min were recently shown not to change resting or 24 h SBP and DBP in untreated hypertensive adults (Kaiser et al., 2025). However, resting blood pressure was reduced in other studies using 36 sessions over 8 weeks of HWI (at 40.5°C) for 90 min in young healthy sedentary people (Brunt et al., 2016), and 30 sessions over 8–10 weeks HWI (at 40.5°C) for 60 min in obese women with polycystic ovary syndrome (Ely et al., 2019). Taken together, these findings suggest that long‐term hypotensive effects might be expected when individual HWI sessions are 60 min or longer and the HWI heat therapy is frequent (four times per week).

We used a 60 min immersion in 40.4°C water to replicate the magnitude of hyperthermia induced by similar heat therapy protocols (Brunt et al., 2016; Ely et al., 2019). Like these previous studies, our participants tolerated the HWI session well, although they all reported a substantial decrease in thermal comfort, and some reported light‐headedness. Therefore, HWI of this duration and severity might be too challenging or unfeasible for some populations. For these populations, an alternative approach might be to combine shorter HWI with exercise, because this is effective in lowering blood pressure over 8–12 weeks in sedentary adults (Steward et al., 2025) and in older adults with peripheral arterial disease (Akerman et al., 2019) and severe lower‐limb osteoarthritis (Roxburgh et al., 2023).

## 5 CONCLUSION

Acute HWI transiently decreased MAP and DBP for ≤20 min post‐heating, whilst SBP was well maintained. In addition, 24 h ABP indicated an increased DBP nocturnal dip, which suggests that HWI has the potential to influence the 24 h blood pressure profile of healthy adults.

## AUTHOR CONTRIBUTIONS

Conception and design of the work: Samuel F. Leaney, Geoff B. Coombs, Jonathan P. Moore and Samuel J. Oliver Acquisition, analysis or interpretation of data for the work: Samuel F. Leaney, Ferrida A. F. Ponce, Tomos F. Owen, Geoff B. Coombs, Benjamin L. Harvey, Oliver N. Davies‐Wilson, Oliver M. A. Brand, Geoff B. Coombs, Jonathan P. Moore and Samuel J. Oliver Drafting the work or revising it critically for important intellectual content: Samuel F. Leaney, Ferrida A. F. Ponce, Tomos F. Owen, Geoff B. Coombs, Benjamin L. Harvey, Oliver N. Davies‐Wilson, Oliver M. A. Brand, Geoff B. Coombs, Jonathan P. Moore and Samuel J. Oliver All authors have read and approved the final version of the manuscript and agree to be accountable for all aspects of the work in ensuring that questions related to the accuracy or integrity of any part of the work are appropriately investigated and resolved. All persons designated as authors qualify for authorship, and all those who qualify for authorship are listed.

## CONFLICT OF INTEREST

The authors declare no conflict of interest.

## Data Availability

The data that support the findings of this study are openly available in figshare, available at https://doi.org/10.6084/m9.figshare.30902018.
